# Cervical rib, case series from a university hospital of Nepal

**DOI:** 10.1016/j.amsu.2021.103061

**Published:** 2021-11-27

**Authors:** Abhyuday Kumar Yadav, Sneha Shrestha, Suyesh Raj Shrestha, Robin Man Karmacharya, Satish Vaidya

**Affiliations:** a Kathmandu University School of Medical Sciences, Dhulikhel, 45210, Nepal; b Unit Chief, Cardio Thoracic and Vascular Surgery Unit, Department of Surgery, Kathmandu University School of Medical Sciences, Dhulikhel, 45210, Nepal

**Keywords:** Case series, Cervical rib, Scalenectomy, Thoracic outlet syndrome

## Abstract

Cervical rib is a rare anatomical anomaly with an incidence of 0.2%–1% and is an important cause of thoracic outlet syndrome. We present a case series of five female patients with a mean age of 20.6 (15–26) years, symptoms present were neck pain, neck mass, tingling sensation and weakness in the affected side. Symptoms develop in adolescence probably due to sagging of the shoulders and a disproportion between chest and neck growth at this age. X-rays of cervical spine was a common mode of diagnosis and showed bilateral cervical rib in three cases and unilateral in two cases. They were managed by performing surgeries under supraclavicular approach with resection of cervical rib of affected side. There was improvement of symptoms with restoration of limb function with a mean time of recovery of 9 weeks. Early diagnosis is important as differential diagnosis of such symptoms may be cervical stenosis and myelopathy which differ in management and have a greater risk of morbidity. In absence of intervention, cervical ribs can lead to compression of neurovascular structures leading to worsening of symptoms, thrombosis of subclavian artery or cerebral emboli.

## Highlights

• Compilation of five cases of cervical ribs which were managed by surgery.
• Details on symptoms and postoperative recovery.
• All the cases were females in age group 15–26 yrs; more on right side.

## 1 Introduction

Cervical ribs are the supernumerary ribs arising mostly from the seventh cervical vertebrae believed to be resulting from mutation of HOX genes.[1] The incidence of cervical ribs in the general population is 0.2%–1.0%.[2], presenting as a complete or incompletely fused bone. Thoracic Outlet Syndrome(TOS) develops from the compression of subclavian artery and the lower trunk of the brachial plexus present within the costoclavicular compartments traversing the interscalene triangle [1]. Symptomatic cervical rib occurs due to compression of neurovascular structures.[3] These symptoms may mimic cervical stenosis possibly leading to inappropriate cervical spine surgery and further increasing complications [3]. (see Table 1)

**Table 1:** Summary of cases of cervical rib.

Case No	Age	Gender	Affected Side	Type	Muscle Power	
					Pre Op	Post Op
1	25	Females	Right (Bilateral)	Type 4	3/5	5/5
2	26	Females	Left (Bilateral)	Type 5	4/5	5/5
3	18	Females	Right (Bilateral)	Type 4	3/5	5/5
4	16	Females	Right	Type 2	4/5	5/5
5	18	Females	Right	Type 4	3/5	5/5

Treatment options include first rib resection with scalenectomy as the operation of choice. There are very few reported cases of cervical ribs in our country. One reported case had thrombosis of the left subclavian artery due to cervical rib. [4] We present a case series of five cervical rib patients improved after surgical resection. This case report was prepared according to PROCESS 2020 criteria.[5].

## 2 Cases

Five patients of Cervical Rib presented to the Cardiothoracic and Vascular Surgery(CTVS) OPD of Dhulikhel Hospital from January 2018 to July 2021. The patients were operated by a team of CTVS surgeons having experiences of 9 yrs and 2 yrs. All the pre operative preparations were carried out according to the standard protocol and optimized accordingly prior to the surgery. All the operations were carried out in the tertiary care university hospital of Nepal within the time span of 3 years. Following surgery, patients were followed up 1 week after discharge, 1 month after discharge and also whenever necessary. This case series has been reported in line with PROCESS 2020 guidelines.[5].

**Fig. 1:**
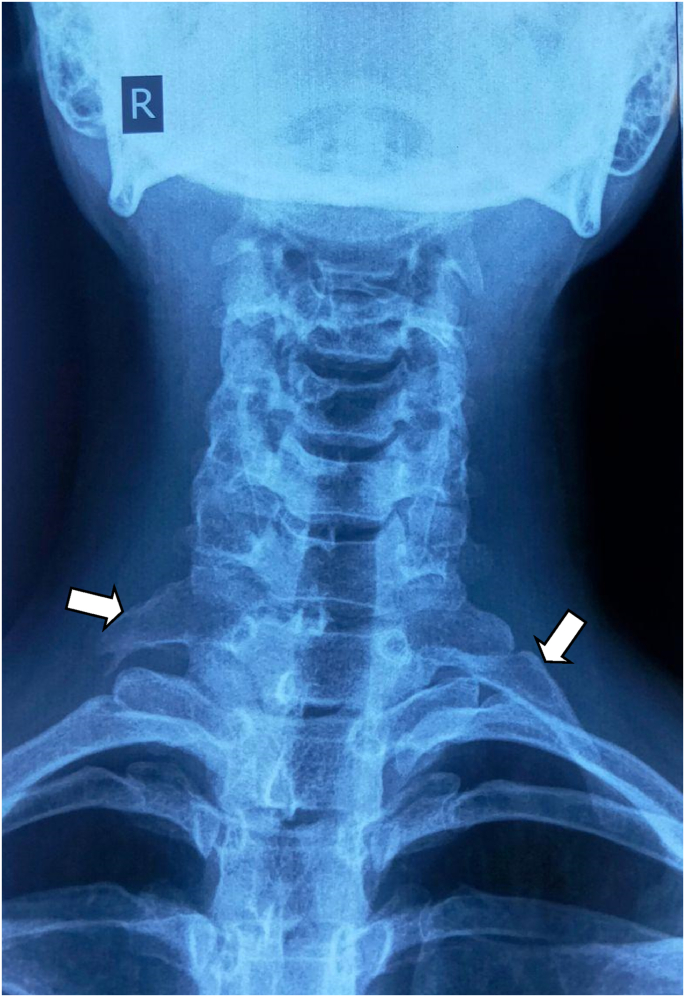
X-ray showing residual right cervical rib (postoperative) and left cervical rib.(shown by arrowhead).

Case 1 25 years female presented with progressive tingling sensation and weakness of right upper arm with muscle power ⅗ for 6 months. She also had shortness of breath for two weeks. Her imaging studies revealed bilateral cervical ribs, more on the right side. Intraoperatively cervical rib type 1 was noted. Following right cervical rib excision, her weakness initially increased for a month after which it gradually reduced. Power of the upper limb returned to normal in two months (see Fig. 1).

**Fig. 2:**
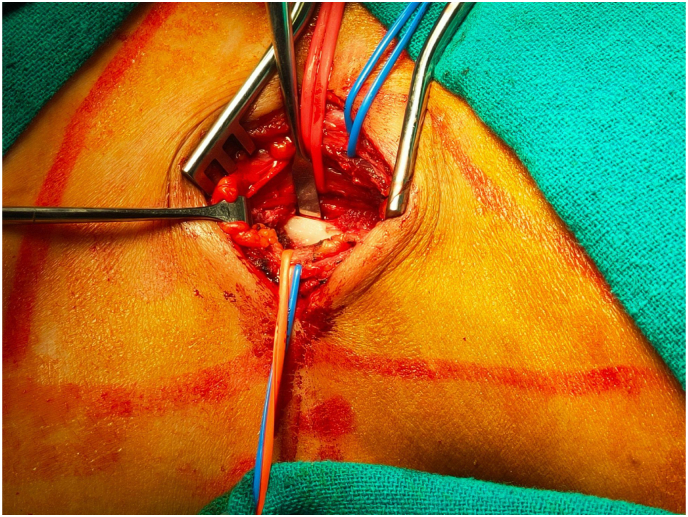
Intraoperative picture before excision of cervical rib.

**Fig. 3:**
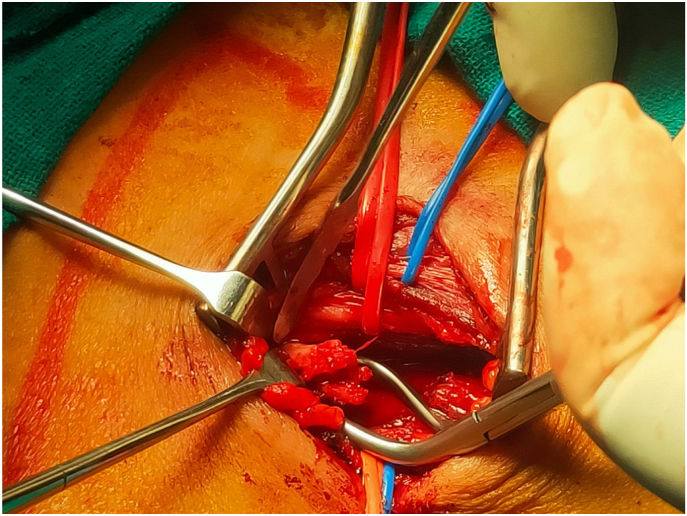
During excision of left cervical rib.

**Fig. 4:**
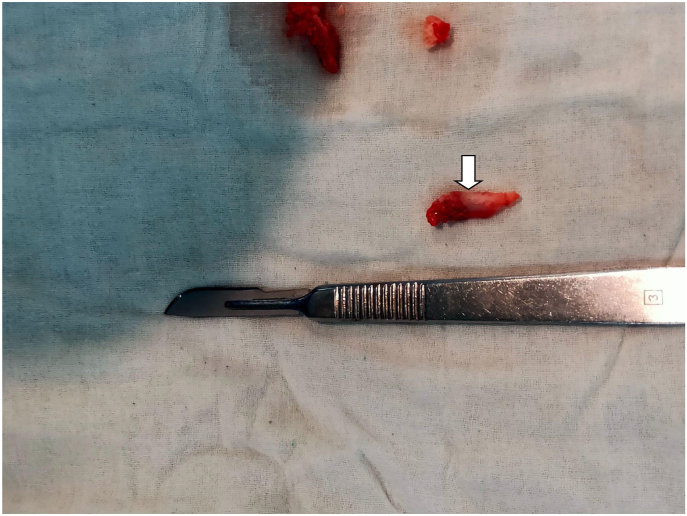
Excised part of left cervical rib.(shown by arrowhead).

Case 2 26 years old female had tingling sensation in left upper arm for two months and weakness of left upper arm with muscle power ⅘. She had a history of right cervical rib excision (Case 1) a year prior. Her current imaging study showed postoperative status of the right cervical rib and presence of left cervical rib. Following the left cervical rib excision, she had improvement of the symptoms which completely resolved in two weeks. Intraoperative picture is shown in Fig. 2 (before excision), Fig. 3 (during excision). The resected part of the rib is shown in Fig. 4.

**Fig. 5:**
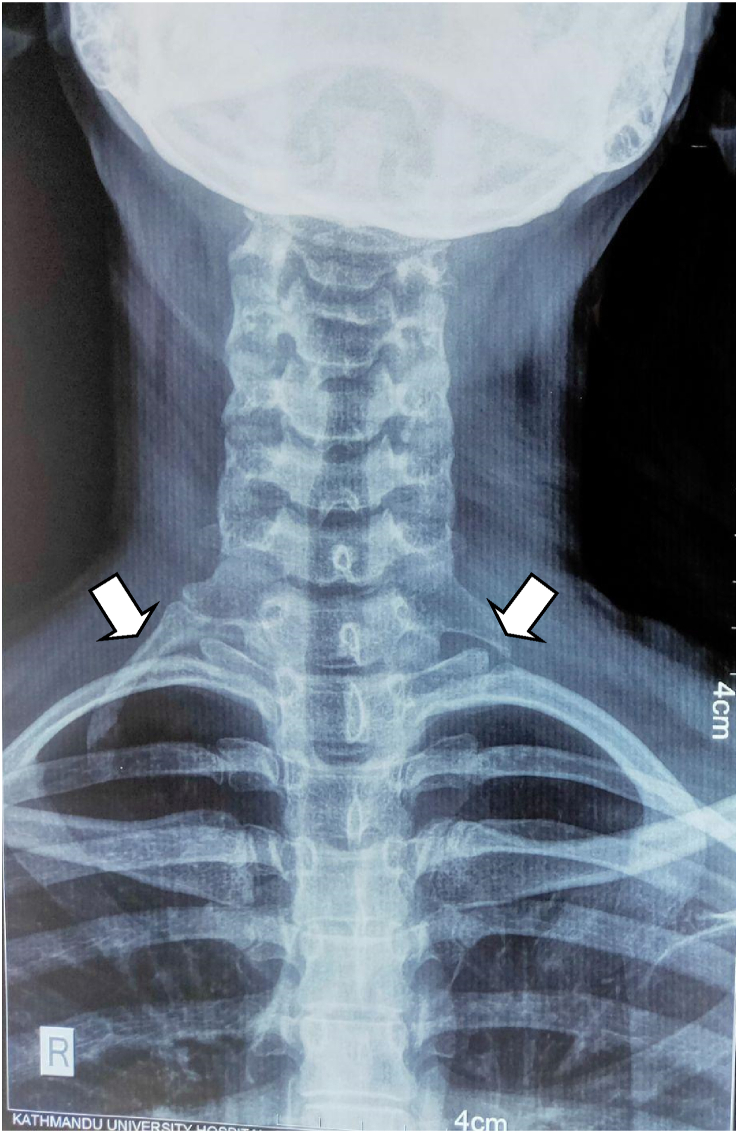
Bilateral cervical rib (right more than left) (shown by arrowhead).

Case3 18 years female with history of numbness and weakness of right upper limb for six months (muscle power ⅗) had bilateral cervical rib (right more than left) as shown in Fig. 5. Right cervical rib excision was done after which the weakness initially increased for two months but gradually resolved. Normal power was regained in six months.

Case4 16 years female from Kathmandu had Right cervical rib causing weakness of right upper limb (muscle power ⅘) for a year and prominent bony lesion with occasional pain in neck. She was diagnosed with Right Cervical Rib fusing with Right first rib posterolaterally forming a pseudoarthrosis. Supraclavicular approach was done under general anesthesia. Following partial resection of omohyoid, identification of brachial plexus was done. Using nibbler and rongers excision of cervical rib was done. Post-operatively, she had weakness for a week after which the power regained. In a month, her power in the right upper limb was completely normal.

**Fig. 6:**
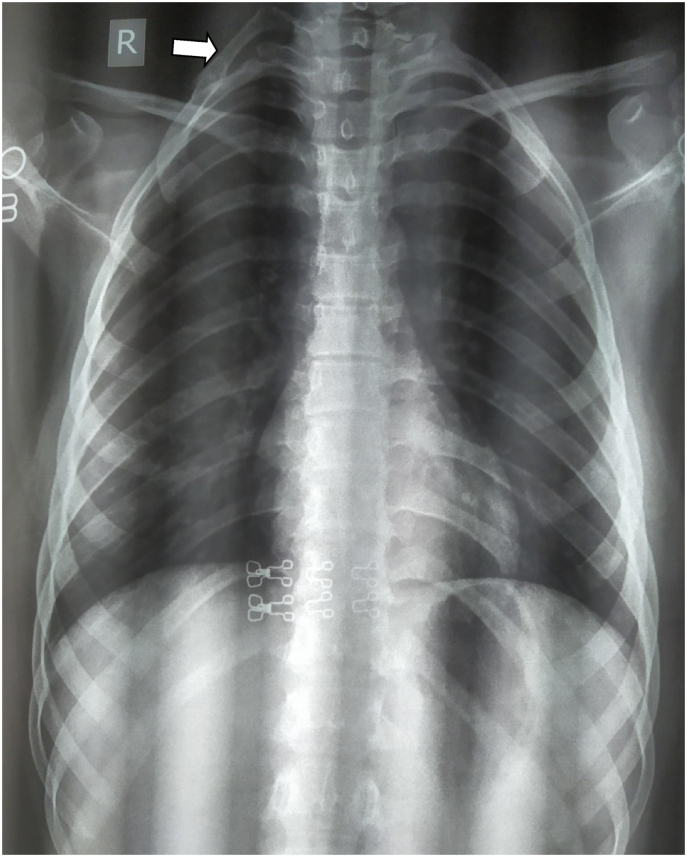
Chest x ray of patient no. 5 showing Right sided Cervical Rib.(Cervical rib shown by arrowhead).

Case5 18 years female from Panchkhal presented with chest pain for 3 years.The pain was in the right upper quadrant of chest involving neck and shoulder as well. The pain was insidious in onset, compressing in character, aggravated on activity and relieved with pain relieving medication.The pain was generally less in morning and maximum at evening. Pain was also associated with tingling sensation in the palm such that she couldn't write or grasp anything. There was also weakness of right upper arm with muscle power ⅗. She was diagnosed with Right sided Cervical Rib (as shown in Fig. 6) with Thoracic Outlet Syndrome. There was no complaint of excessive hiccups. Supraclavicular approach was done under general anesthesia. Brachial plexus, phrenic nerve, omohyoid muscles were retracted. Both proximal and distal ends were separated and the distal end was made free. Hemostasis was maintained and drain was kept. Muscles and subcutaneous tissue were closed with vicryl. Skin was sutured with prolene 3–0 and cotton bandage was applied. Per operative findings were 6 cm*2 cm*1 cm sized Right cervical rib extending from C7 with its anterior end attached to the clavicle.

In summary, all the patients in our series were female with a mean age of 20yrs. Mean duration of symptoms was 1 year. Mean duration to regain power in the affected limb was 9 weeks. Right side was affected in four cases while the left side was affected in one case.

## 3 Discussion

Cervical ribs are prolongations of the transverse processes of the seventh cervical vertebrae and are usually attached to the first rib.[6,7] In the early fetal period, lateral costal processes along the vertebrae form ribs which arise from precursor sclerotome cells in the thoracic region. Continued migration of sclerotome precursor cells in the cervical region may lead to formation of cervical rib.[3,4] The size of the cervical rib is not the index to the symptoms. Halstead was the first to report that when an artery is subjected to incomplete pressure an aneurysm develops distal to the point of pressure.[8] Todd advanced a theory of the nervous origin of vascular changes in the hands suggesting that the changes in the arteries were due to pressure causing paralysis of the sympathetic fibers. Irritation of these fibres induces spasm of the arterial wall, obliteration of the vasa vasorum; ultimately leading to thrombosis and occlusion.[9] In 1869, Gruber proposed a classification of cervical rib based on the amount of bone present and the thickness of the rib-like structure which was later modified by Blanchard. This classification is divided into five types.3.

In Type 1, Complete cervical rib is attached to the sternum. In Type 2, Cartilage of the cervical rib is attached to cartilage of the first rib. In Type 3, the two extremities of the ribs are developed as bone structures, but the intermediate portion is a fibrous cord. In Type 4 the two extremities are developed but not united by a fibrous cord, in Type 5 cervical rib is represented by a segment attached to the vertebrae, no anterior extremity exists.[3] In our series three cases were of type 4 cervical rib, one was of type 2 (case 4) and one case of type 5 cervical rib (Case 2).

Almost 90% of the cases of cervical rib are asymptomatic and do not require any treatment. When symptomatic, cases can have either neurological and vascular manifestations the type of manifestation depends on the morphology of the cervical ribs with incomplete ribs only affecting the brachial plexus, whereas complete ribs also affect the subclavian artery.[10].

Neurological manifestation includes pain, paresthesia and numbness in the affected arm mainly on the ulnar side, difficulty in holding pen. Vascular manifestation includes pain, weakness,discoloration of arm, diminished pulses at wrist, prolonged capillary refill time, pulsatile neck mass.[10] Cerebral embolus is a rare but noted complication as a result of retrograde flow from subclavian artery compression. Of the five cases, three had bilateral presentations. According to a study performed in 2013 by Sharma et al., on 5000 chest radiograms of residents from India Cervical ribs were more commonly unilateral (0.78%) than bilateral (0.44%)[10]. Symptoms are common in the right due to more frequent use of the right, more dropping of the right shoulders in right-handed persons, and also due to the close proximity of the right plexus to the right rib.[3] The deformity is more common in women than men in a ratio of 2:1. Women present more often with symptoms than men.[11] Female preponderance is attributed to the greater movement of the chest in women or deformities being noted more often by women. [3].

In our case series, all the patients were female with a mean age of 20, the index patient is a woman. Our patient presented with pain,weakness, mainly in the right hand and also neck mass on the right side, and with the breathing problem due to nerve compression by cervical rib. Even though this disease is congenital it manifests generally at the mean age of 30 years probably due to sagging of shoulders with increasing age which puts more traction on the neurovascular structure giving rise to symptoms.[3].

Symptomatic patients need to undergo treatment. Treatment can be carried out either conservatively or surgically. Multimodal treatment approach including patient education, modified physical activity(targeted muscle strengthening) and pharmacological treatment comes under conservative mode of management. [12] Analgesics, muscle relaxants, anticonvulsants, injection of local anesthetic are some of the drugs that provide symptomatic relief. Surgical management is another mode of treatment depending upon the type of thoracic outlet syndrome(neurogenic, venous or arterial) carried out when the conservative methods don't benefit the patient. The conservative method of management in neuronal type thoracic outlet syndrome(nTOS) should last for 4–6 months before surgery; the surgery of choice being first rib resection and scalenectomy or scalenotomy.[13] The particular mode of treatment among the different methods for cervical rib resection(Transaxillary, supraclavicular and infraclavicular) is based on the individual patient and their anatomy.[14] Video Assisted Thoracoscopic Surgery, robotic-assisted and endoscopic-assisted *trans*-axillary approaches are the newly advanced surgical options. Early treatment of the symptomatic cervical rib, even if there is a shorter duration of symptoms, is essential for the better end result. Prolonged duration of symptoms may result in deterioration of the symptoms and reduced functionality.[15].

Certain studies have shown brachial plexus injury post first rib resection. Brachial plexus injuries were reported in 0.6% patients with nTOS following transaxillary first rib resection and 4% incidence after supraclavicular first rib resection.[14] Other late complications of TOS surgery include the recurrence of symptoms, phrenic nerve paralysis, subclavian artery and vein injuries, hemothorax, pneumothorax, weakness of the hand muscles, sensory deficits and autonomic dysfunction.[16].

In our case, surgery was done under Supraclavicular approach. This is the most preferred approach and was also done in most of the patients included under the research performed by Henry et al.[1]. Time taken for recovery was 9 weeks as compared to 12 weeks as reported by Cu et al.[3].

Our case series including 5 patients showed that cervical ribs can have multiple presentations. Although the average period of recovery was 9 weeks here, this can vary from patient to patient. Surgical treatment in this case is complex. However, outcomes will be good given that the cases are properly managed.

## 4 Conclusion

Cervical rib, though a rare anatomical anomaly commonly presents with symptoms of neck pain and tingling sensation of hands leading to a possible differential diagnosis. Early diagnosis and interventions reduce the risk of further complications leads to better prognosis.

## Provenance and peer review

Not commissioned, externally peer-reviewed.

## Sources of funding

Since we are medical students under supervision and we have just started doing the research, we don't have any financial support for our research.

## Ethical approval

As the case series is compilation of information of retrospective period, we had obtained exempt for ethical approval from Institutional ethical committee.

## Consent

Informed written consent was taken from all the cases for inclusion of the case details in this case series. We also ensured, none of the identifying characteristics are included in the case series.

## Author contribution

Please specify the contribution of each author to the paper, e.g. study concept or design, data collection, data analysis or interpretation, writing the paper, others, who have contributed in other ways, should be listed as contributors.

## Registration of research studies

1. Name of the registry:
2. Unique identifying number or registration ID:
3. Hyperlink to your specific registration (must be publicly accessible and will be checked):

## Guarantor

Dr. Robin Man Karmacharya, Associate Professor and unit chief, Cardio Thoracic and Vascular Surgery Unit, Department of Surgery, Dhulikhel Hospital Kathmandu University Hospital.

## Declaration of competing interest

There are no conflicts of interest.
