# Supplementary material for: Cervical rib, case series from a university hospital of Nepal
Source: Ann Med Surg (Lond). 2021 Nov 27;72:103061. doi: 10.1016/j.amsu.2021.103061 (PMC8636984; doi:10.1016/j.amsu.2021.103061)
Supplement: Multimedia component 1 [file mmc1.docx]

| **PROCESS 2020 Checklist** | | | |
| --- | --- | --- | --- |
| **Topic** | **Item** | **Checklist Item Description** | **Page Number** |
| **Title** | **1** | - **Cervical Rib, Case Series From a University Hospital of Nepal.** | 1 |
| **Key Words** | **2** | - Case series, Cervical rib, Females, Thoracic outlet syndrome | 2 |
| **Abstract** | **3a** | Introduction and Importance   - Cervical rib is a rare anatomical anomaly with an incidence of 0.2% to 1% and is an important cause of thoracic outlet syndrome. - We present a case series of five female patients with a mean age of 20 (15- 26) years. | 2 |
|  | **3b** | Methods   - Not relevant | 2 |
|  | **3c** | Outcomes   - Describe the outcomes of the intervention and management strategy. - Cervical rib is more common in females and on one side, mainly the right side. Surgical intervention under Supraclavicular approach is the most preferable management strategy. | 2 |
|  | **3d** | Conclusion   - Cervical rib, though a rare anatomical anomaly commonly presents with symptoms of neck pain and tingling sensation of hands leading to a possible differential diagnosis. Early diagnosis and interventions reduce the risk of further complications leads to better prognosis. | 15 |
| **Introduction** | **4** | - Cervical ribs are the supernumerary ribs arising mostly from the seventh cervical vertebrae as a transverse process believed to be resulting from mutation of HOX genes.[^1^](https://paperpile.com/c/6ovHmY/mICU/?noauthor=1) The incidence of cervical ribs in the general population is 0.2% to 1.0%.[^2^](https://paperpile.com/c/6ovHmY/wrfS), presenting as a complete or incompletely fused bone. Cervical ribs are mostly asymptomatic or give rise to Thoracic Outlet Syndrome. Thoracic Outlet Syndrome(TOS) develops from the compression of subclavian artery and the lower trunk of the brachial plexus present within the costoclavicular compartments traversing the interscalene triangle[^1^](https://paperpile.com/c/6ovHmY/mICU). Symptomatic cervical rib occurs due to compression of neurovascular structures.[^3^](https://paperpile.com/c/6ovHmY/4WpL)These  symptoms  may mimic  cervical  stenosis possibly leading   to  inappropriate  cervical  spine  surgery and further increasing complications[^3^](https://paperpile.com/c/6ovHmY/4WpL). - Treatment options include first rib resection with scalenectomy as the operation of choice. There are very few reported cases of cervical ribs in our country. One reported case had thrombosis of the left subclavian artery due to cervical rib. [^5^](https://paperpile.com/c/6ovHmY/PCWK) This case report was prepared according to SCARE 2020 criteria. [^6^](https://paperpile.com/c/6ovHmY/Podj) | 2,3 |
| **Methods** | **5a** | Registration   - Not relevant. | - |
|  | **5b** | Study Design   - The case study is a case series. - The case series is retrospective. |  |
|  | **5c** | Settings and Time-Frames   - The patient was managed in the Dhulikhel Hospital which is community based. The patient was followed up 1 week post surgery, 1 month post surgery and also when necessary. |  |
|  | **5d** | Participants   - The patients of Dhulikhel Hospital are the participants in this case series. |  |
|  | **5e** | Pre-Intervention Patient Optimisation   - The standard protocols for surgery under general anesthesia had been carried out. |  |
|  | **5f** | Interventions   - Surgical interventions were carried out for the treatment of this condition. - Analgesics were also prescribed to the patients. |  |
|  | **5g** | Intervention Details.   - Surgery was performed under general anesthesia with patient in supine position. Prolene 3-0 was used for suturing |  |
|  | **5h** | Operator Details   - The procedure was done by a team of thoracic surgeons with experience from 2 to 9 years. |  |
|  | **5i** | Quality Control   - All the cases were diagnosed, treated and followed by the same team. |  |
|  | **5j** | Follow-Up   - Following discharge the patient was followed up in three, seven, 14 days. After that they were followed up every monthly. |  |
| **Results** | **6a** | Participants   - There are a total of five patients in this case series. None of them have any significant comorbidities. | 11 |
|  | **6b** | Deviation from the Initial Management Plan   - NA |  |
|  | **6c** | Outcomes and Follow-Up   - The details are explained. | 12,13 |
|  | **6d** |  |  |
|  | **6e** | - Postoperative issues are explained. |  |
| **Discussion** | **7a** | - Summarise the key results. | 6 |
|  | **7b** | Relevant Literature and Placing the Results in Context   - Include a discussion of the relevant literature and, if appropriate, similar published studies. - Describe the implications for clinical practice guidelines (e.g. NICE) and any relevant hypotheses generated. | 6 |
|  | **7c** | Strengths   - Describe the relevant strengths of the study. - Detail any multidisciplinary or cross-speciality relevance.   Weaknesses and Limitations   - Describe the relevant weaknesses or limitations of the study. - For novel techniques or devices, outline any contraindications and alternatives, potential risks and possible complications if applied to a larger population. | 6 |
|  | **7d** | Directions for Future Research   - State how the methodology and findings discussed can impact future research and clinical practice. Describe the questions that have arisen as a result of this study. - State the alternative study design(s) best suited to address these questions. | 7 |
| **Conclusions** | **8a** | Key Conclusions   - Outline the key conclusions from this study. | 8 |
|  | **8b** | Rationale   - Ensure that any of the conclusions made are supported by a strong rationale. | 8 |
|  | **8c** | Future Work   - Briefly discuss any questions arisen from this study and any differences in approach to patient diagnosis or management which the authors might adopt in future similar studies. | 8 |
| **Patient Perspective** | **9** | - Where appropriate, the patients should be given the opportunity to share their perspective on the intervention(s) they received (e.g. sharing quotes from a consented, anonymised interview, or questionnaire). | - |
| **Informed Consent** | **10** | - Informed written consent was taken from all the cases for inclusion of the case details in this case series. We also ensured, none of the identifying characteristics are included in the case series. | - |
| **Additional Information** | **11a** | - There are no conflicts of interest. | - |
|  | **11b** | - Since we are medical students under supervision and we have just started doing the research, we don’t have any financial support for our research. | - |
|  | **11c** | - | - |
| **Clinical Images and Videos** | **12** | - Where relevant and available, include clinical images to help demonstrate the cases pre-, peri-, and post-intervention (e.g. radiological, histopathological, patient photographs, intraoperative images). - Where relevant and available, include a link (e.g. Google Drive, YouTube) to the narrated operative video to highlight specific techniques or operative findings. - Ensure all media files are appropriately captioned and indicate points of interest to allow for easy interpretation. | 4,5,6,7,8,10 |
| **Referencing the Checklist** | **13** | - Include reference to the PROCESS 2020 publication by stating: 'This case series has been reported in line with the PROCESS Guideline' at the end of the methods section (and include citation in the references section). | 15 |
